# The relationship between meaningful activity and apathy among assisted living residents with dementia

**DOI:** 10.1002/alz.086603

**Published:** 2025-01-09

**Authors:** Jennifer Klinedinst

**Affiliations:** ^1^ University of Maryland Baltimore, Baltimore, MD USA

## Abstract

**Background:**

Apathy, or reduction in goal directed behaviors, is common in assisted living (AL) residents with dementia. Meaningful activity, defined as participation in activities that are enjoyable, tailored to the individual’s interests and abilities, related to a personally relevant goal, engaging, and related to an aspect of an individual’s identity, may be a promising strategy for reducing apathy in AL residents with dementia. However, there is a dearth of evidence examining the relationship between engagement in meaningful activity and apathy among AL residents with dementia. The purpose of this study is to determine the relationship between engagement in meaningful activity and apathy among AL residents with dementia. We hypothesized that AL residents who have apathy would have lower levels of engagement in meaningful activity than those residents without apathy.

**Method:**

This study was a secondary data analysis using baseline data from a randomized controlled trial, Meaningful Activity for Managing Behavioral Symptoms of Distress (MAC‐4‐BSD) of 71 residents from 5 AL communities in Maryland. Apathy was measured using the apathy subscale of the Neuropsychiatric Inventory Questionnaire (NPI‐Q). Meaningful activity was measured from the resident using the Engagement in Meaningful Activities Survey (EMAS). Descriptive statistics including means or frequencies were conducted to describe the sample. A one‐tailed independent samples t‐test was conducted to test the hypothesis.

**Result:**

Participants were on average 85.39 (s.d. = 8.20) years old, with moderate to severe dementia (Saint Louis University Mental Status exam score average m = 6.76, s.d. = 6.06). The majority were female (n = 52) and White (n = 62). They had an average length of stay of 1.84 years (s.d. = 1.67) and 8.24 (s.d. = 2.62) comorbidities. Overall, 41% (n = 29) of the participants had apathy. The mean EMAS score was significantly lower (37.52, s.d. = 10.54) among those with apathy than among those without apathy (41.63, s.d. = 9.62) (t = 1.69, p<.05).

**Conclusion:**

Individuals living with dementia in AL with apathy tend to spend less time engaged in meaningful activities. Interventions are needed to test if increasing engagement in activities that are considered meaningful by the resident can help reduce apathy in this population and thereby improve quality of life.

